# SAFE–IOERT prospective study: clinical outcomes of exclusive intraoperative radiotherapy in early breast cancer according to molecular subtype and tumor-infiltrating lymphocytes

**DOI:** 10.1007/s12094-026-04445-y

**Published:** 2026-06-08

**Authors:** G. Oses, C. Clavell, B. González-Farré, E. Sanfeliu, M. Laplana, A. Martínez, S. Moreno, G. Meca, E. Gomis, J. Carreras-Ballvé, E. Mension, I. Cebrecos, X. Caparrós, J. Tarrats-Rosell, C. Cases, A. Herreros, M. Mollà

**Affiliations:** 1 Department of Radiation Oncology, Hospital Clínic of Barcelona, Barcelona, Spain; 2 Department of Pathology, Hospital Clínic of Barcelona, Barcelona, Spain; 3 Department of Gynecology, Hospital Clínic of Barcelona, Barcelona, Spain; 4 Faculty of Medicine, University of Barcelona, Barcelona, Spain; 5 Translational Genomics and Targeted Therapies in Solid Tumors Group, August Pi I, Sunyer Biomedical Research Institute (IDIBAPS), Barcelona, Spain

**Keywords:** Early breast cancer, IOERT, APBI, Local recurrence, Adverse effects, TILs

## Abstract

**Purpose and objectives:**

Intraoperative electron radiotherapy (IOERT) can be applied during breast conserving surgery to treat invasive breast cancer. Tumor-infiltrating lymphocytes (TILs) are fundamental elements of the specific immunological response against tumor cells and have prognostic importance in many types of cancer. The aim of the study was to analyze the local recurrence rate, adverse effects, surrogate molecular subtype, and stromal percentage of TILs in patients with breast cancer treated with exclusive IOERT.

**Materials and methods:**

Eighty-one patients with early-stage breast carcinoma were included in a prospective study and were treated performing a conservative surgery, followed by IOERT with a dose of 20 Gy as accelerated partial breast irradiation (APBI). TILs were evaluated in hematoxylin–eosin sections of surgical specimens before IOERT.

**Results:**

The median follow-up of the patients was 58 months (range 15–128). Fifty-eight patients (71.6%) were classified as Luminal A-like, 21 patients (25.9%) Luminal B-like and 2 patients (2.5%) were classified as HER2 positive-like. The local recurrence rate was 2.5%. Two patients presented ipsilateral local recurrence, and one patient presented distant recurrence. Eight patients (9.9%) suffered chronic toxicity with grade 1 localized fibrosis, fat necrosis, and pain. Eighty-one percent of stromal TILs were low and 14.9% were intermediate. In 3 patients (4.1%) the stromal percentage of TILs could not be assessed.

**Conclusions:**

In this prospective cohort of carefully selected patients with early breast cancer, exclusive IOERT delivered as accelerated partial breast irradiation achieved favourable local control with minimal chronic toxicity. Predominantly low stromal TIL levels in luminal-like tumours may be associated with recurrence risk. Larger prospective studies are needed for validation.

## Introduction

Intra Operative Radiation Therapy (IORT) is a single-fraction treatment with low-energy photons or electrons (IOERT) of a surgically exposed area. In the targeted region, it can inhibit the proliferative capacity of residual tumor cells or eliminate microscopic tumor foci during surgery. This approach also protects surrounding organs at risk by maintaining physical distance from the radiation field or using adequate lead shielding. Moreover, IORT also increases the likelihood that the tumor bed will be accurately identified without the restrictions of imaging exams.

IOERT can be used in breast carcinoma after breast-conserving surgery, under clinical trial, as accelerated partial breast irradiation (APBI) in patients with low risk of locoregional recurrence [1]. In accordance with international guidelines, APBI seems to be a good treatment option for properly selected low-risk breast cancer. Criteria according to APBI guidelines: stage I/II breast cancer, non-lobular, unicentric and unifocal, with negative margins after surgery, positive receptor status, with a tumor ≤ 3 cm, and a sentinel lymph node with 0–3 positive nodes without extracapsular lymph node metastasis (ECE) [1–3].

Estrogen receptor-positive (ER +)/human epidermal growth factor receptor 2 (HER2 −) breast cancer is a heterogeneous disease, in terms of biology, clinical characteristics and prognosis. Such heterogeneity affects response to treatments. Luminal A tumors, as by molecular intrinsic subtyping, are known to be more sensitive to endocrine treatment as compared to Luminal B tumors, which may benefit from adding chemotherapy [4]. These molecular subtypes are usually the ones selected for treatment with exclusive IOERT, more specifically the luminal A-like subtype.

Tumor-infiltrating lymphocytes (TILs), located in the tumor microenvironment, are the fundamental elements of the specific immunological response against tumor cells and have prognostic importance in many types of cancer [5, 6]. Research involving thousands of patients has explored the prognostic role of TILs in breast cancer, and their relevance depends on the biologic subtype [7, 8]. TILs are quantified as a percentage of the stromal area occupied by mononuclear inflammatory cells, following International Immuno-Oncology Biomarker Working Group guidelines [9].

IOERT began to be used in our center in 2013, all patients were included in a prospective study, after approval by the hospital ethics committee. We conducted a prospective review of our institutional experience with APBI–IOERT in patients with early-stage breast cancer. We describe the recurrence rate, adverse effects, surrogate molecular subtypes, and the stromal percentage of TILs. To the best of our knowledge, this is the first report to evaluate stromal TILs in patients with early-stage breast carcinoma treated with IOERT. The analysis of stromal TILs in these breast cancer subtypes is relevant, as it may provide further insight into recurrence patterns.

## Materials and methods

### Patient and tumor characteristics

In 2013 a prospective study was approved by the hospital ethics committee (record: 2013/8864). We analyzed 81 patients who underwent surgery and IOERT between May 2013 and April 2023. All the patients signed an informed consent form and were selected and discussed in a multidisciplinary oncologic board. The indications for IOERT in our series were: age ≥ 50 years; ductal and other favorable histologies; unicentric and unifocal; positive receptor status; pN0 (i-/i +); grade 1/2; tumor size ≤ 2 cm. Were treated performing a conservative surgery with intraoperative sentinel node assessment, followed by IOERT with a dose of 20 Gy as APBI. TILs were evaluated in hematoxylin–eosin sections of surgical pieces before IOERT. Clinical outcomes including local recurrence, adverse effects, surrogate molecular subtypes and stromal TIL percentages were prospectively evaluated.

### Treatment characteristics

Patients were initially treated via surgery combined with adjuvant therapies depending on the characteristics of each individual case. The results of salvage surgical procedures were classified by pathological anatomy intraoperatively before IOERT in: R0, free surgical margins; R1, resection with focally microscopically involved margins; and R2, visible or palpable residual tumor. Characteristics of the patients and previous treatment to IOERT are shown in Table 1.

**Table 1:** Basal characteristics and adverse effects

Histology	N	%
Invasive ductal carcinoma	69	85.2
Invasive lobular carcinoma	5	6.2
Ductal carcinoma in situ	1	1.2
Invasive mucinous carcinoma	2	2.5
Invasive tubular carcinoma		
Invasive papillary carcinoma	1	1.2
	3	3.7
*Histological grade*		
1	37	45.7
2	42	51.9
3	2	2.5
*Stage*		
pT1aN0	3	3.7
pT1bN0	27	33.3
pT1cN0	40	49.3
pT2N0	11	13.6
*Subtype*		
Luminal A-like	58	71.6
Luminal B-like	21	25.9
HER2 positive-like	2	2.5
Triple negative-like	0	0
*Stromal percentage TILs*		
Low (0–10%)	66	81
Intermediate (11–40%)	12	14.9
High (41–90%)	0	0
Unknown	3	4.1
*Chemotherapy*		
Yes	2	2.5
No	79	97.5
*Hormone therapy*		
Yes	76	93.8
No	5	6.2
*Adherence to hormone therapy*		
Yes		
No	61	75.3
	20	24.7
*Acute adverse effects *	*N*	*%*
Minor seroma	14	17.3
Localized edema	3	3.7
Minor hematoma	3	3.7
*Chronic adverse effects*	*N*	*%*
Grade 1 fat necrosis	4	4.9
Grade 1 fibrosis	1	1.2
Grade 1 localized pain	3	3.7

A dedicated 10 MeV mobile electron linear accelerator (LIAC) (S.I.T. Sordina IORT Technologies S.p.A., Italy) was used to provide IOERT. The electron beam was delivered through transparent PMMA (polymethylmethacrylate) cylindrical applicators with different diameters (3 to 10 cm). To spare underlying tissues from radiation, a shielding disk—available in various diameters—was always placed. The protective disc consists of a steel disc that is inserted in a PTFE (Polytetrafluoroethylene) sleeve. The orientation of the disc is such that the sleeve is facing upward (toward the tumor bed) to efficiently shield secondary electrons backscattered and avoid undesired overdosage of the superjacent tissue. The most common applicator diameter was 5 cm (range 3–7), and only flat tubes were used. The applicator size was chosen in order to ensure the proper coverage for a given target volume around the surgical sutured breech, depending on the tumor size and location. We defined the Planning Target Volume as a perimeter expansion of 2 cm beyond the former macroscopic tumor edge. The most used energy of the electron beams was 6 MeV (range 4–10), and it was chosen according to the depth of the tumor bed. The applicator was placed directly in contact with the target volume. The median prescribed dose was 20 Gy (range 18–20).

### Study endpoints and statistical analysis

The study endpoints of local recurrence (LR), regional recurrence (RR), distant recurrence (DR) were evaluated. LR was calculated between the date of IOERT and the date of the first in-IOERT field recurrence. RR was defined from the date of IOERT to the date of first outside-IOERT field recurrence within the anatomical site. DR was measured from the date of IOERT to the date of the first recurrence outside. Acute and chronic toxicities attributable to IOERT were scored according to Common Terminology Criteria for Adverse Events (CTCAE) version 5.0 (grades 1–5). Stromal tumor-infiltrating lymphocytes (TIL) were quantified as a percentage of the stromal area occupied by mononuclear inflammatory cells, following International Immuno-Oncology Biomarker Working Group guidelines [9]. TILs were morphologically evaluated by pathologists specialized in breast carcinoma on hematoxylin and eosin-stained slides from the surgical specimens. The mean, standard deviation, median, range and frequencies of the prognostic variables were analyzed. The local recurrence rate was analyzed with the Kaplan–Meier method and the log-rank test was used for contrasting results, considering the *p* value of < 0.05 bilaterally significant. The statistical analysis was carried out through the IBM SPSS version 25.0.

## Results

The median follow-up of the patients was 58 months (range 15–128). The median age was 76 years (range 53–94). The median energy used in the IOERT was 6 MeV (range 4–10). Sixty-nine patients (85.2%) were diagnosed with invasive ductal carcinoma and the other 12 patients (14.8%) were diagnosed with other subtypes (tubular, mucinous and papillary carcinoma). All patients had free surgical margins (R0). Seventy patients (86.4%) were staged as pT1pN0 and 11 patients (13.6%) as pT2pN0. Fifty-eigth patients (71.6%) were classified as Luminal A-like, 21 patients (25.9%) Luminal B-like and 2 patients (2.5%) were classified as HER2 positive-like (Table 1).

**Fig. 1:**
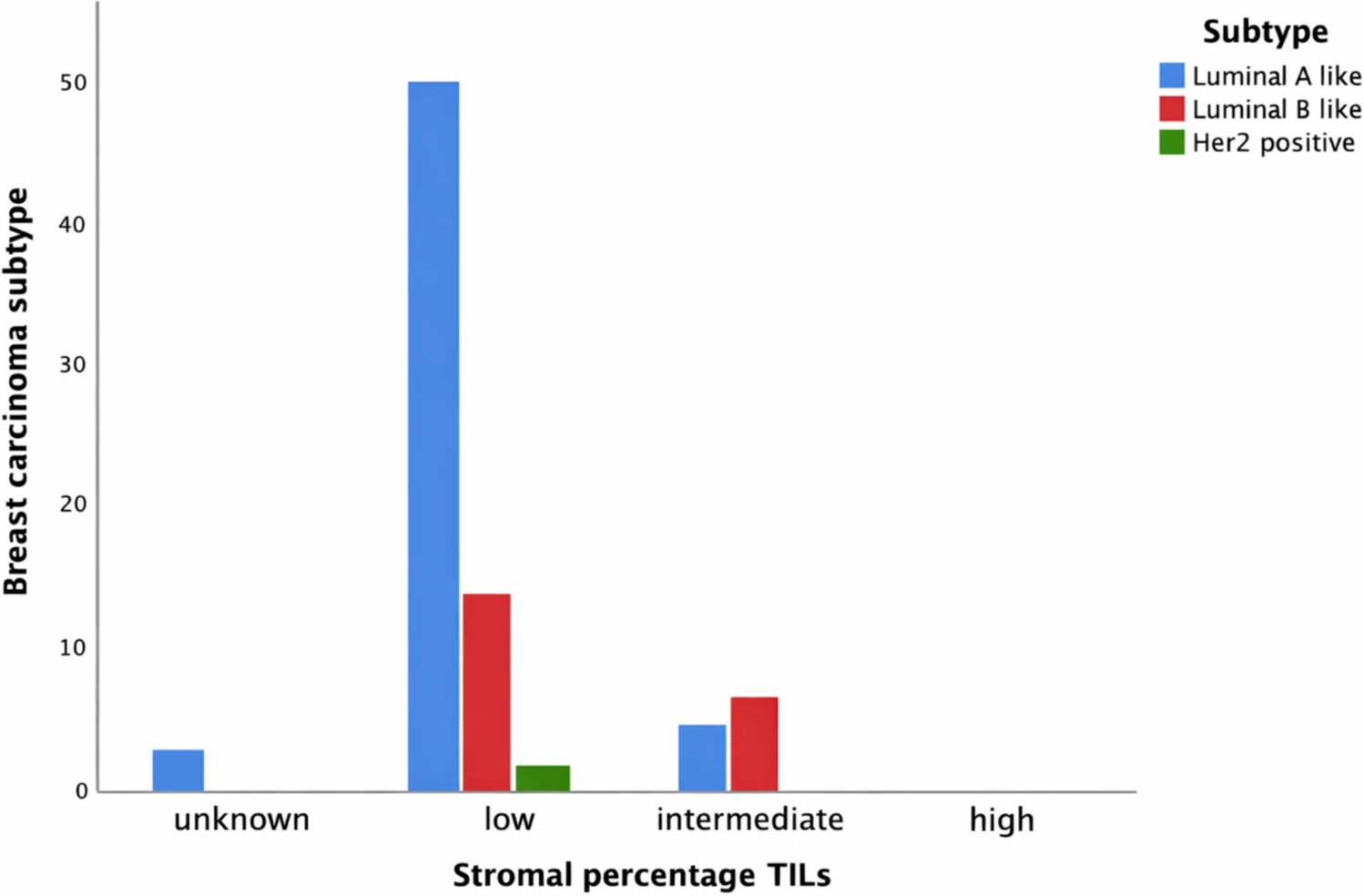
Association between breast carcinoma subtype and stromal percentage of TILs

The median TILs in the stromal tumoral was 5% (Q1–Q3 2.25–10). Eighty-one percent of stromal TILs were low and 14.9% were intermediate. In 3 patients (4.1%) the stromal percentage of TILs could not be assessed. Fifty-nine percent (48) of Luminal A-like patients had low stromal TILs, 6.1% (5) had intermediate stromal TILs. Twenty percent (16) of Luminal B-like patients had low stromal TILs, 8.6% (7) had intermediate stromal TILs. Two point four percent (2) of HER2 positive-like patients had low stromal TILs. Patients with Luminal A and Luminal B-like subtypes mostly presented a low stromal percentage of TILs (Fig. 1).

Twenty patients (24.7%) presented acute toxicity in form of edema, seroma and hematoma. Only 8 patients (9.9%) suffered chronic toxicity grade 1 with localized fibrosis, fat necrosis, and pain (Table 1). Seventy-six patients (93.8%) underwent adjuvant hormonal therapy and only 2 patients (2.5%) underwent adjuvant chemotherapy. The median adherence to hormone therapy was 48 months (1–120). The local recurrence rate was 2.5%. Two patients developed ipsilateral breast tumor recurrence. One patient experienced recurrence at 2 years and 7 months, which was treated with mastectomy. The tumor was luminal A-like, and the TIL level was 10%. The second patient had recurrence at 5 years and 5 months; the tumor was luminal B-like, with a TIL level of 20%. This patient underwent lumpectomy followed by external beam radiotherapy, receiving a total dose of 40.05 Gy in 15 fractions. In addition, one patient developed distant recurrence. The tumor was luminal B-like, with a TIL level of 5%, and presented with pulmonary metastases. At the final control, 80.3% of the patients (65 patients) are alive and 19.7% (16 patients) have died. In 1 patient the cause was cancer specific and in the remaining 15 patients it was due to another cause.

## Discussion

In this study, we prospectively evaluated local control, survival outcomes, and toxicity, as well as quantified the percentage of stromal TILs and assessed their association with breast carcinoma subtypes in 81 patients who underwent breast-conserving surgery followed by APBI with IOERT. To our knowledge, this is the first study to specifically investigate stromal TILs in patients treated with IOERT. The rational for IOERT, compared to other APBI techniques, is that it is administered immediately after tumor removal in a way that surgery and radiation therapy are completed in a one-step session. The intra-operative direct view of the tumor bed lowers the risk of geographic miss, the skin receives a lower radiation dose and organs at risk also receive a reduced dose, contributing to better cosmetic outcome [10]. While some groups have reported the presence of misalignments between the placement of the applicator and the protective disk using live verifications with radiochromic films, improvement of the setup could lead to better results in terms of irradiation of the organs at risk [11].

However, literature data points out that careful patient selection for IOERT is mandatory and should be offered to patients at low risk of ipsilateral breast tumor recurrence (IBTR). In the ELIOT trial, a phase III randomized study comparing IOERT with whole-breast irradiation (WBI) for early breast cancer, the ipsilateral local recurrence rate was 4.4% at 5 years with IOERT versus 0.4% with WBI [12]. In longer-term follow-up, the cumulative recurrence rates in the IOERT group were 4.2%, 8.1%, and 12.6% at 5, 10, and 15 years, respectively, compared with 0.5%, 1.1%, and 2.4% in WBI group [1]. The ELIOT trial included a considerable proportion of higher risk tumours (pN positive, histological grade 3, and triple-negative subtypes), whereas this study strictly focused on a low risk population (age ≥ 50 years, positive hormone receptors, pN0, histological grade 1–2, tumor size ≤ 2 cm, unicentric disease and favourable biology). Notably, in the the ELIOT trial’s predefined very low‑risk subgroup (tumor size < 1 cm, histological grade 1, luminal A-like subtypes, Ki‑67 < 14%), comprising 141 patients, ipsilateral local recurrence rate remained low with both treatment modalities. IOERT showing 5‑, 10‑, and 15‑year ipsilateral local recurrence rate of 0%, 1.3%, and 8.1%, compared with 0%, 1.5%, and 3.1% for WBI (HR 1.97, p = 0.45). These findings strengthens our assertion that proper selection is the major determinant of outcome and supports the rationale for exclusive IOERT in biologically favourable tumors [1]. Regarding overall survival, long-term analyses of the ELIOT trial demonstrated no significant differences between IOERT and WBI, indicating that although local recurrence may be slightly higher with IOERT, survival outcomes are comparable. Our results, showing a low 5-year local recurrence rate closely resembling that of WBI, further support the notion that IOERT is a safe and effective technique when patients are carefully selected. In our cohort, only two ipsilateral local recurrences were observed, both occurring within the first six years after treatment. Although the median follow-up was 58 months, the low number of events and the limited number of patients at risk beyond 10 years preclude robust estimation of long-term actuarial local recurrence rates comparable to those reported in the ELIOT trial. Overall, these data highlight the crucial role of rigorous selection criteria to minimize recurrence risk [1, 12].

In 2024, a meta-analysis was published that included 18 retrospective, prospective, and randomized studies (2010–2013), including 5483 patients with breast carcinoma treated with IOERT. Thirteen studies reported 203 local recurrences. Nine studies reported 47 nodal recurrences. Four papers reported 35 ipsilateral second breast cancers. Thirteen papers described 436 recurrences of any type. The pooled proportion of 5-year probability of local recurrence, only reported in 4 papers, was 6% (95% CI 3–9%). They concluded that despite the heterogeneity of the studies and that there is only one randomized trial, IOERT seems a feasible option for appropriately selected patients. They also pointed out the fact that current phase I/II or retrospective/prospective studies have reported lower ipsilateral local recurrence rates compared to the ELIOT trial [13]. A letter to the editor was subsequently published, highlighting that, given the heterogeneity of the studies included in this meta-analysis, additional prospective data are needed [14].

In this study, with 58 months of median follow-up and with a careful selection of patients, the rate of loco-regional recurrence was 2.5% (only two patients presented local recurrence, undergoing treatment with endocrine therapy). Twenty patients (24.7%) presented minor acute toxicity in form of edema, seroma and hematoma. Only 8 patients (9.9%) had chronic toxicity grade 1 with localized fibrosis, fat necrosis, and pain. Our results are in correlation with some of the prospective trials included in the meta-analysis. Sawaki M et al. conducted a multicenter, phase II study, analyzing 129 patients undergoing IOERT and after a mean follow-up of 59.5 months, the ipsilateral local recurrence rate rate was 3.1% (95% confidence interval: 0.9–7.8). Toxicities included grade 1 fibrosis in deep connective tissue in 78.1% of patients; grade 3 and grade 2 wound infections were observed in 1.6% of patients, respectively, and grade 3 and grade 2 soft tissue necrosis occurred in 0.8% of patients, respectively [15]. On the other hand, Kawamura M et al. developed a prospective phase I/II trial of IOERT, including 32 patients, with a mean follow-up of 10 years, they did not present recurrences in the treatment field, only two patients presented a regional recurrence at 8 years. CTCAE Grade 2 fibrosis was experienced by three patients as an acute adverse event and by two patients as a late adverse event. There were no additional ≥ Grade 2 late adverse events, including hematoma, infection, pain or dermatitis [16]. Philippson et al. prospectively enrolled 996 patients, with a median follow-up of 71.9 months, presented a local recurrence rate of 3.5%. Acute treatment toxicity was low, with 1.5% hematomas, 0.8% infections, 0.9% wound dehiscence, 0.6% having both an infection and wound dehiscence, 1% with delayed uncomplicated wound closure, and 0.1% of wound necrosis. Late toxicities were not indicated for the entire set of patients given that those assessments were progressively discontinued [17]. Although these studies are not randomized, it has been shown that after a correct selection of patients to undergo IOERT, with follow-up between 5 and 10 years, the locoregional recurrence rates are lower.

This study represents, to the best of our knowledge, the first detailed description of stromal TILs in a cohort of breast cancer patients treated with IOERT. Although the results obtained do not allow us to establish a causal association between lymphocytic infiltration and the administration of IOERT, the analysis of the tumor immune microenvironment may help generate hypotheses regarding the potential mechanisms involved in local response and tumor control. It is well-established that the prognostic relevance of TILs depends on the biologic subtype of breast cáncer [7, 8]. Patients with triple-negative breast cancer (TNBC) display a robust, linear relationship between increased number of stromal TILs and disease-free survival (DFS), as noted in multiple retrospective and prospective analyses [7, 18]. A similar association was found for patients with HER2 positive tumors [19]. Overall, the studies strongly support the integration of TILs in a clinico-pathologic prognostic model for patients with TNBC and HER2 + breast cancer. However, the prognostic and predictive role of TILs in ER +/HER2 − breast cancer remains a subject of debate [20, 21]. ER +/HER2 − tumors tend to have a scarce immune infiltrate. In a large analysis of patients treated with neoadjuvant therapy for ER +/HER2 − breast cancer, higher TILs correlated with higher pathological complete response (pCR) rate to chemotherapy, but also higher TILs were associated with poorer outcome in this specific subtype, in contrast to what we usually observe in other subtypes of breast cancer [21, 22]. According to published studies, high TIL levels in ER +/HER2 − breast cancer treated in the adjuvant setting have been significantly associated with clinicopathological features linked to unfavorable outcomes [6]. To date, no studies have specifically characterized stroal TILS in patients treated with IOERT. In our cohort, 59% (48) of Luminal A-like patients had low stromal TILs, 6.1% (5) had intermediate stromal TILs. Twenty percent (16) of Luminal B-like patients had low stromal TILs, 8.6% (7) had intermediate stromal TILs. Two point four percent (2) of HER2 positive-like patients had low stromal TILs. Our results correspond with the literature, in patients with luminal A-like and luminal B-like subtypes, who present a low stromal percentage of TILs, have a good prognosis. In addition, this may be associated with low risk of local recurrence. This study, therefore, contributes new data by characterizing stromal TILs within a carefully selected luminal cohort treated with exclusive IOERT, providing preliminary observations that may help generate hypotheses about the tumor immune microenvironment in this setting. Patients with HER2-positive and triple-negative breast cancer were under-represented in our cohort, mainly because these biological subtypes are commonly excluded from eligibility criteria for partial breast irradiation strategies. Therefore, the exploratory findings regarding molecular subtype and stromal TILs should be interpreted cautiously and cannot be generalized beyond carefully selected luminal-like populations. Prospective clinical trials and standardized methodologies are needed to validate the prognostic or predictive role of TILs in luminal breast cancer and to define clinically meaningful cutoff thresholds.

### Limitations to study

Our series includes a limited number of patients, and longer follow-up might be necessary, as locoregional recurrences of breast carcinoma can occur beyond 5 years. Patients with luminal B-like, HER2-positive, and triple-negative breast carcinomas are under-represented in the cohort, largely because these subtypes are frequently excluded from eligibility criteria for partial breast irradiation. IOERT was administered after surgical tumor resection, meaning the tumor cells were not directly irradiated. As a result, the assessment of TILs was not conducted on paired samples (biopsy and surgical specimen).

## Conclusions

In this prospective cohort of carefully selected patients with early breast cancer, exclusive IOERT delivered as accelerated partial breast irradiation achieved favourable local control with minimal chronic toxicity. Predominantly low stromal TIL levels in luminal-like tumours may be associated with recurrence risk. Larger prospective studies are needed for validation.

## References

[1] Orecchia R, Veronesi U, Maisonneuve P, Galimberti VE, Lazzari R, Veronesi P, et al. Intraoperative irradiation for early breast cancer (ELIOT): long-term recurrence and survival outcomes from a single-centre, randomised, phase 3 equivalence trial. Lancet Oncol. 2021;22(5):597–608. 10.1016/S1470-2045(21)00080-2. 33845035

[2] Fastner G, Gaisberger C, Kaiser J, Scherer P, Ciabattoni A, Petoukhova A, et al. ESTRO IORT task force/ACROP recommendations for intraoperative radiation therapy with electrons (IOERT) in breast cancer. Radiother Oncol. 2020;149:150–7. 10.1016/j.radonc.2020.04.059. (**Epub 2020 May 13. PMID: 32413529**). 32413529

[3] Shaitelman SF, Anderson BM, Arthur DW, Bazan JG, Bellon JR, Bradfield L, et al. Partial breast irradiation for patients with early-stage invasive breast cancer or ductal carcinoma in situ: an ASTRO clinical practice guideline. Pract Radiat Oncol. 2024;14(2):112–32. 10.1016/j.prro.2023.11.001. 37977261

[4] Prat A, Fan C, Fernández A, et al. Response and survival of breast cancer intrinsic subtypes following multi-agent neoadjuvant chemotherapy. BMC Med. 2015;13:303. 10.1186/s12916-015-0540-z. PMC4683815 26684470

[5] Salgado R, Denkert C, Demaria S, Sirtaine N, Klauschen F, Pruneri G, et al. International TILs Working Group 2014. The evaluation of tumor-infiltrating lymphocytes (TILs) in breast cancer: recommendations by an International TILs Working Group 2014. Ann Oncol. 2015;26(2):259–71. 10.1093/annonc/mdu450. PMC6267863 25214542

[6] Criscitiello C, Vingiani A, Maisonneuve P, et al. Tumor-infiltrating lymphocytes (TILs)in ER+/HER2− breast cancer. Breast Cancer Res Treat. 2020;183:347–54. 10.1007/s10549-020-05771-7. 32621251

[7] Loi S, Sirtaine N, Piette F, Salgado R, Viale G, Van Eenoo F, et al. Prognostic and predictive value of tumor-infiltrating lymphocytes in a phase III randomized adjuvant breast cancer trial in node-positive breast cancer comparing the addition of docetaxel to doxorubicin with doxorubicin-based chemotherapy: BIG 02-98. J Clin Oncol. 2013;31(7):860–7. 10.1200/JCO.2011.41.0902. (**Epub 2013 Jan 22. PMID: 23341518**). 23341518

[8] Ali HR, Provenzano E, Dawson SJ, Blows FM, Liu B, Shah M, et al. Association between CD8+ T-cell infiltration and breast cancer survival in 12,439 patients. Ann Oncol. 2014;25(8):1536–43. 10.1093/annonc/mdu191. (**Epub 2014 Jun 9 PMID: 24915873**). 24915873

[9] Salgado R, Denkert C, Demaria S, Sirtaine N, Klauschen F, Pruneri G, et al. The evaluation of tumor-infiltrating lymphocytes (TILs) in breast cancer: recommendations by an International TILs Working Group 2014. Ann Oncol. 2015;26(2):259–71. 10.1093/annonc/mdu450. (**Epub 2014 Sep 11. PMID: 25214542; PMCID: PMC6267863**). PMC6267863 25214542

[10] Wenz F, Blank E, Welzel G, Hofmann F, Astor D, Neumaier C, et al. Intraoperative radiotherapy during breast-conserving surgery using a miniature x-ray generator (Intrabeam®): theoretical and experimental background and clinical experience. Women’s Health Lond. 2012;8(1):39–47. 10.2217/whe.11.82. (**PMID: 22171773**). 22171773

[11] Cases C, Oses G, Herreros A, Tarrats-Rosell J, Moreno S, Mollà M. On the need of in vivo verifications as quality control for intraoperative electron radiotherapy in breast cancer. Clin Transl Oncol. 2024;26(7):1623–9. 10.1007/s12094-023-03378-0. (**Epub 2024 Jan 24. PMID: 38267659**). 38267659

[12] Veronesi U, Orecchia R, Maisonneuve P, Viale G, Rotmensz N, Sangalli C, et al. Intraoperative radiotherapy versus external radiotherapy for early breast cancer (ELIOT): a randomised controlled equivalence trial. Lancet Oncol. 2013;14(13):1269–77. 10.1016/S1470-2045(13)70497-2. (**Epub 2013 Nov 11. PMID: 24225155**). 24225155

[13] Aristei C, Camilli F, Epifani V, Borghesi S, Palumbo I, Bini V, et al. A systematic review and meta-analysis of intraoperative electron radiation therapy delivered with a dedicated mobile linac for partial breast irradiation in early breast cancer. Breast. 2024;76:103759. 10.1016/j.breast.2024.103759. (**Epub 2024 Jun 4. PMID: 38851057; PMCID: PMC11219955**). PMC11219955 38851057

[14] Maisonneuve P, Orecchia R. Meta-analysis of intraoperative electron radiation therapy for partial breast irradiation in early breast cancer. Breast. 2024;77:103787. 10.1016/j.breast.2024.103787. (**Epub 2024 Aug 15. PMID: 39154488; PMCID: PMC11381795**). PMC11381795 39154488

[15] Sawaki M, Miyamoto T, Fujisawa T, Itoh Y, Ebara T, Tachibana H, et al. Multicenter Phase II study of intraoperative radiotherapy of early breast cancer: Ipsilateral tumor recurrence. Ann Surg Oncol. 2019;26(8):2428–34 (**Epub 2019 Apr 16. PMID: 30993410**). 10.1245/s10434-019-07350-7 30993410

[16] Kawamura M, Itoh Y, Kamomae T, Sawaki M, Kikumori T, Tsunoda N, et al. A phase I/II trial of intraoperative breast radiotherapy in an Asian population: 10-year results with critical evaluation. J Radiat Res. 2020;61(4):602–7. 10.1093/jrr/rraa029. (**PMID: 32494808; PMCID: PMC7336551**). PMC7336551 32494808

[17] Philippson C, Larsen S, Simon S, Vandekerkhove C, De Caluwe A, Van Gestel D, et al. Intraoperative electron radiotherapy in early invasive ductal breast cancer: 6-year median follow-up results of a prospective monocentric registry. Breast Cancer Res. 2022;24(1):83. 10.1186/s13058-022-01582-4. (**PMID: 36419161; PMCID: PMC9685863**). PMC9685863 36419161

[18] Loi S, Michiels S, Salgado R, Sirtaine N, Jose V, Fumagalli D, et al. Tumor infiltrating lymphocytes are prognostic in triple negative breast cancer and predictive for trastuzumab benefit in early breast cancer: results from the FinHER trial. Ann Oncol. 2014;25(8):1544–50. 10.1093/annonc/mdu112. (**Epub 2014 Mar 7. PMID: 24608200**). 24608200

[19] Salgado R, Denkert C, Campbell C, Savas P, Nuciforo P, Aura C, et al. Tumor- infiltrating lymphocytes and associations with pathological complete response and event-free survival in HER2-Positive early-stage breast cancer treated with lapatinib and trastuzumab: a secondary analysis of the NeoALTTO trial. JAMA Oncol. 2015;1(4):448–54. 10.1001/jamaoncol.2015.0830. (**Erratum in: JAMA Oncol. 2015 Jul;1(4):544. doi: 10.1001/jamaoncol.2015.1805. Erratum in: JAMA Oncol. 2015 Nov;1(8):1172. doi: 10.1001/jamaoncol.2015. 4229. Nucifero, Paolo [corrected to Nuciforo, Paolo]. Erratum in: JAMA Oncol. 2019 Jan 1;5(1):122. doi: 10.1001/jamaoncol.2018.5678. PMID: 26181252; PMCID: PMC5551492**). PMC5551492 26181252

[20] Dieci MV, Griguolo G, Miglietta F, Guarneri V. The immune system and hormone receptor positive breast cancer: is it really a dead end? Cancer Treat Rev. 2016;46:9–19. 10.1016/j.ctrv.2016.03.011. (**Epub 2016 Mar 28. PMID: 27055087**). 27055087

[21] Denkert C, von Minckwitz G, Darb-Esfahani S, Lederer B, Heppner BI, Weber KE, et al. Tumour-infiltrating lymphocytes and prognosis in different subtypes of breast cancer: a pooled analysis of 3771 patients treated with neoadjuvant therapy. Lancet Oncol. 2018;19(1):40–50. 10.1016/S1470-2045(17)30904-X. (**Epub 2017 Dec 7. PMID: 29233559**). 29233559

[22] Stanton SE, Disis ML. Clinical significance of tumor-infiltrating lymphocytes in breast cancer. J Immunother Cancer. 2016;4(1):59. 10.1186/s40425-016-0165-6. (**PMID: 27777769; PMCID: PMC5067916**). PMC5067916 27777769

